# Supplementary figures and images for: Amino acid variants of SARS-CoV-2 papain-like protease have impact on drug binding
Source: PLoS Comput Biol. 2022 Nov 21;18(11):e1010667. doi: 10.1371/journal.pcbi.1010667 (PMC9721480; doi:10.1371/journal.pcbi.1010667)

Percentage of mutated sequences  
according to month

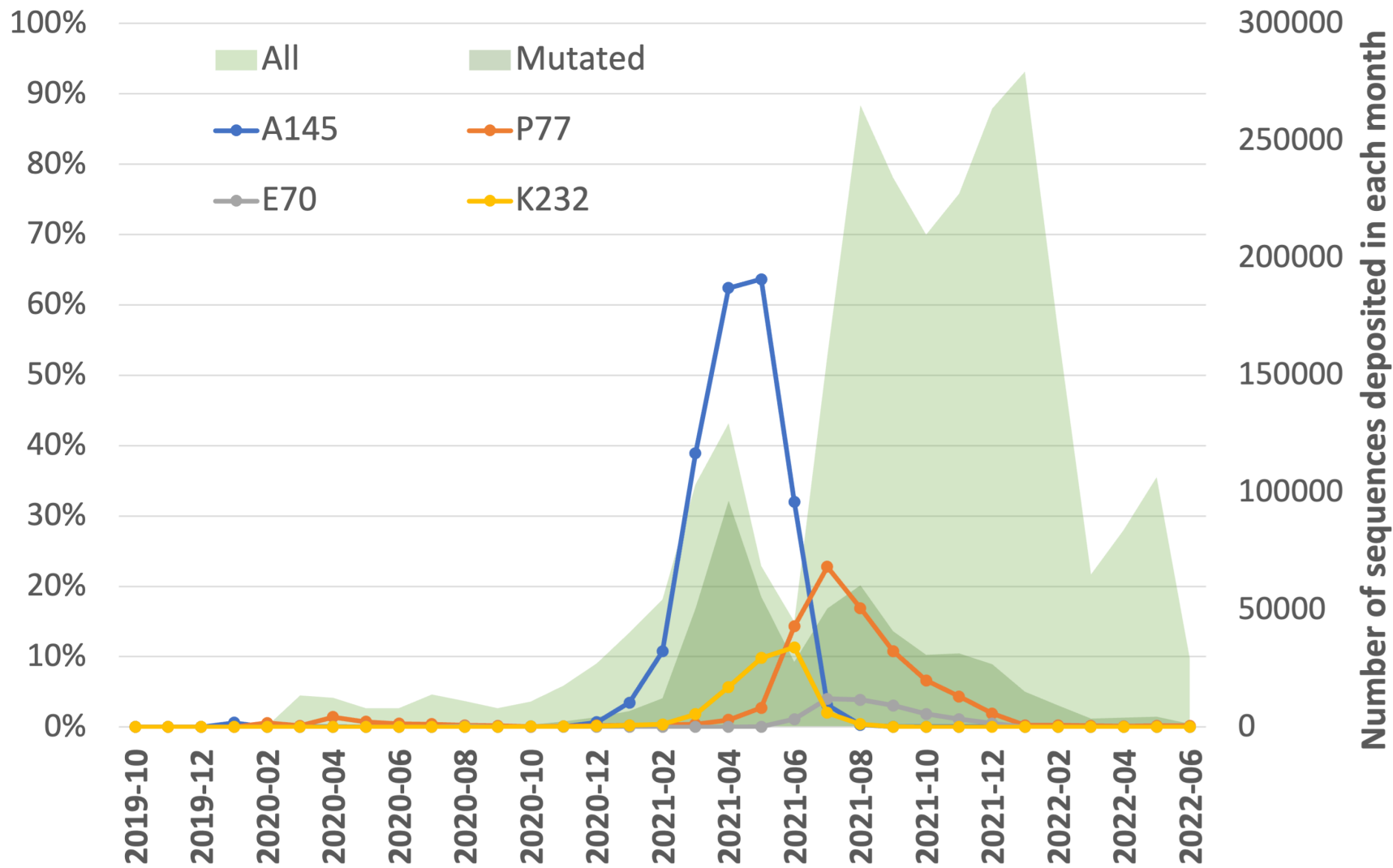

Supplement: S1 Fig — Lines show distribution of the sequences carrying the most frequent variants. For example, in April and May 2021 more than 60% of the mutated sequences had a modification on 145 position. Shaded areas show number of deposited PLpro sequences—light green show all of the sequences, dark green only those carrying at least one mutation. From April to June 2021 the majority of the deposited sequences had at least one variant. (PDF) [file pcbi.1010667.s001.pdf]

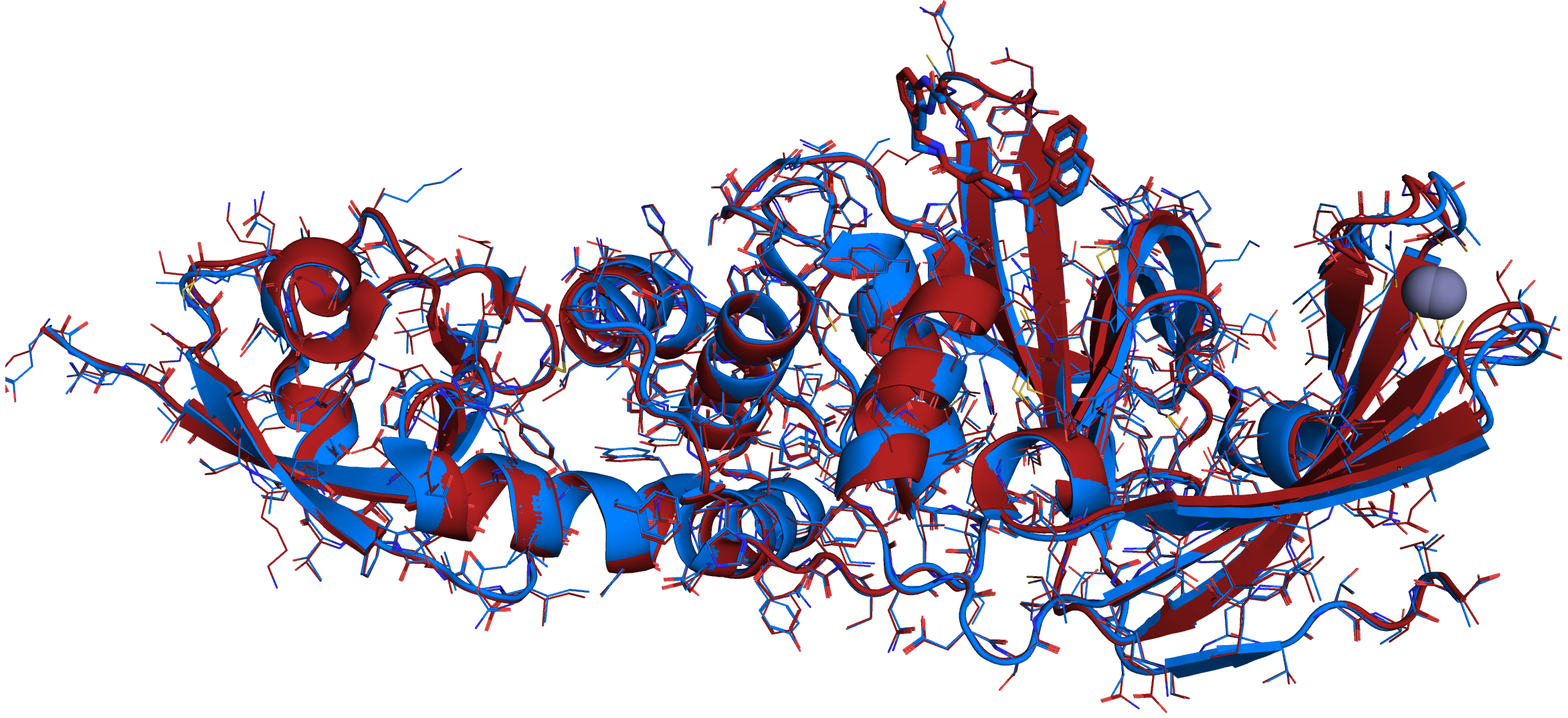

Supplement: S2 Fig — Based on PDB IDs 7d7t (red; removed from PDB) and 7e35 (blue; supersedes 7d7t). Cα RMSD is 0.4 Å, RMSD of all heavy atoms is 0.8 Å. (TIF) [file pcbi.1010667.s002.tif]

C-alpha RMSD (Å)

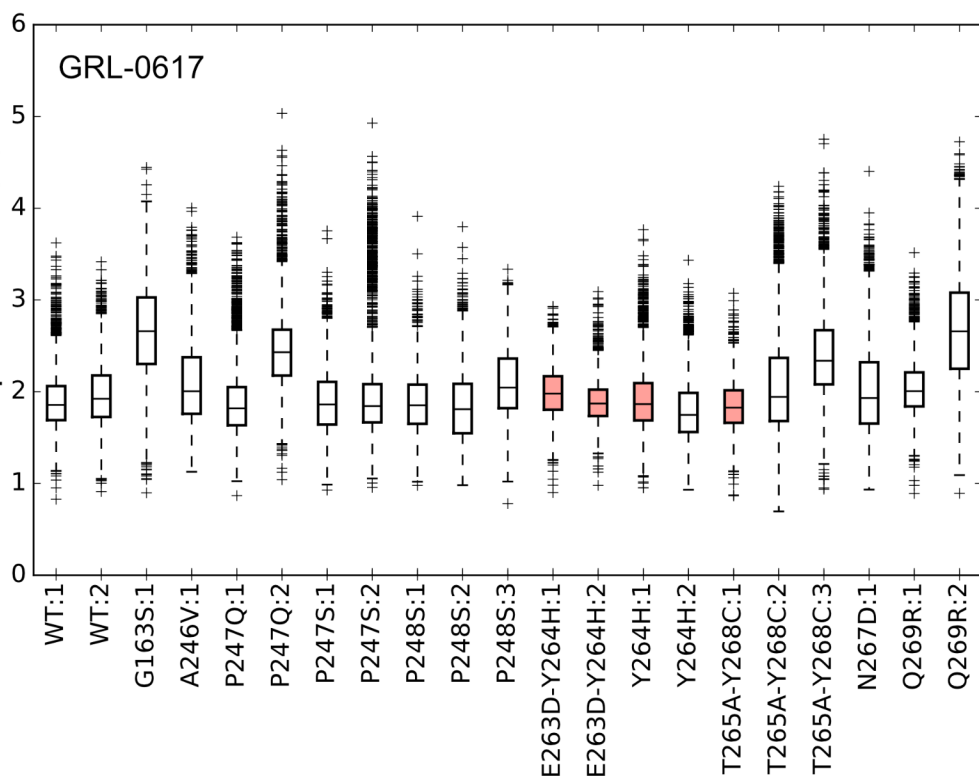

C-alpha RMSD (Å)

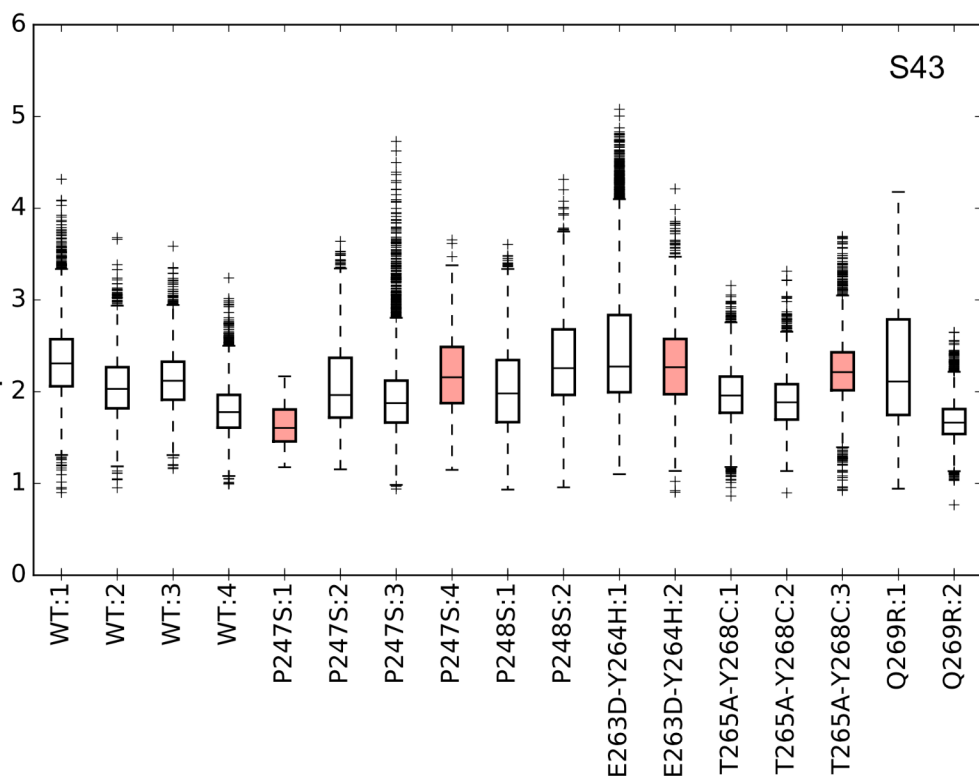

Supplement: S3 Fig — Left: GRL-0617, right: S43. The trajectories in which the ligand dissociated from the binding site are marked with red—the length of these trajectories is truncated to the time the ligand stayed in the site (see S1 Table). (PDF) [file pcbi.1010667.s003.pdf]

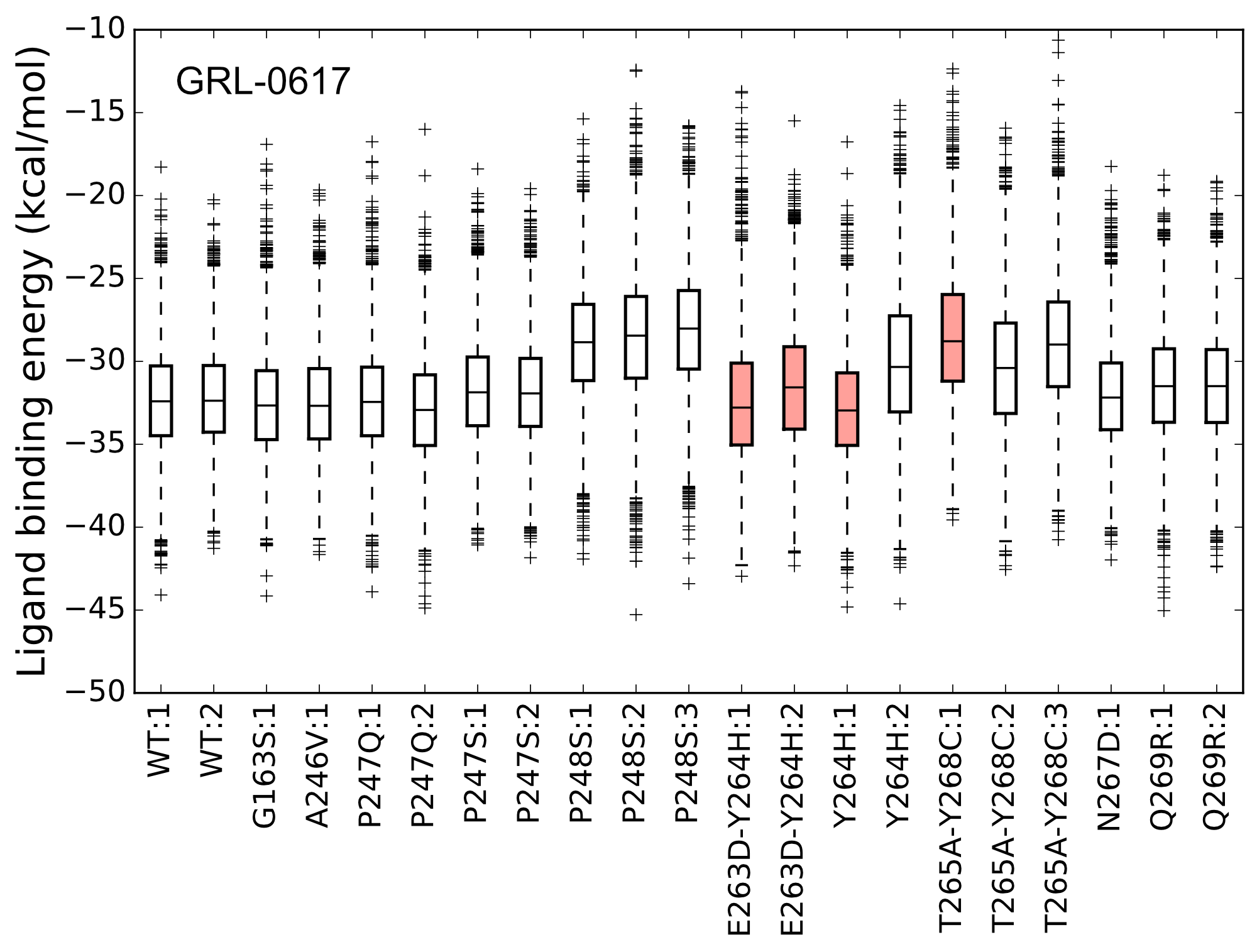

Supplement: S4 Fig — The calculations are based on MMGBSA method. The trajectories in which the ligand dissociated from the binding site are marked with red—the length of these trajectories is truncated to the time the ligand stayed in the site (see S1 Table). (TIF) [file pcbi.1010667.s004.tif]

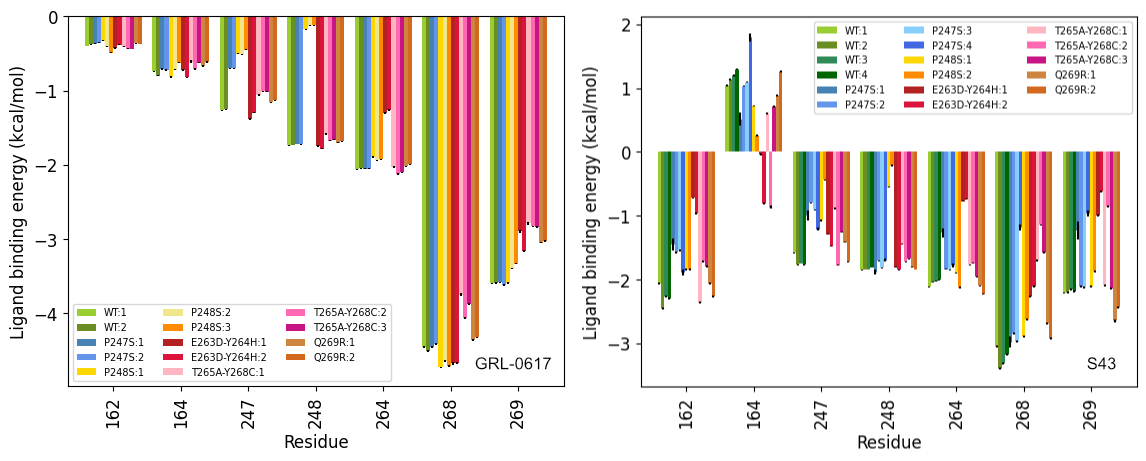

Supplement: S5 Fig — The calculations are based on MMGBSA method. (TIF) [file pcbi.1010667.s005.tif]

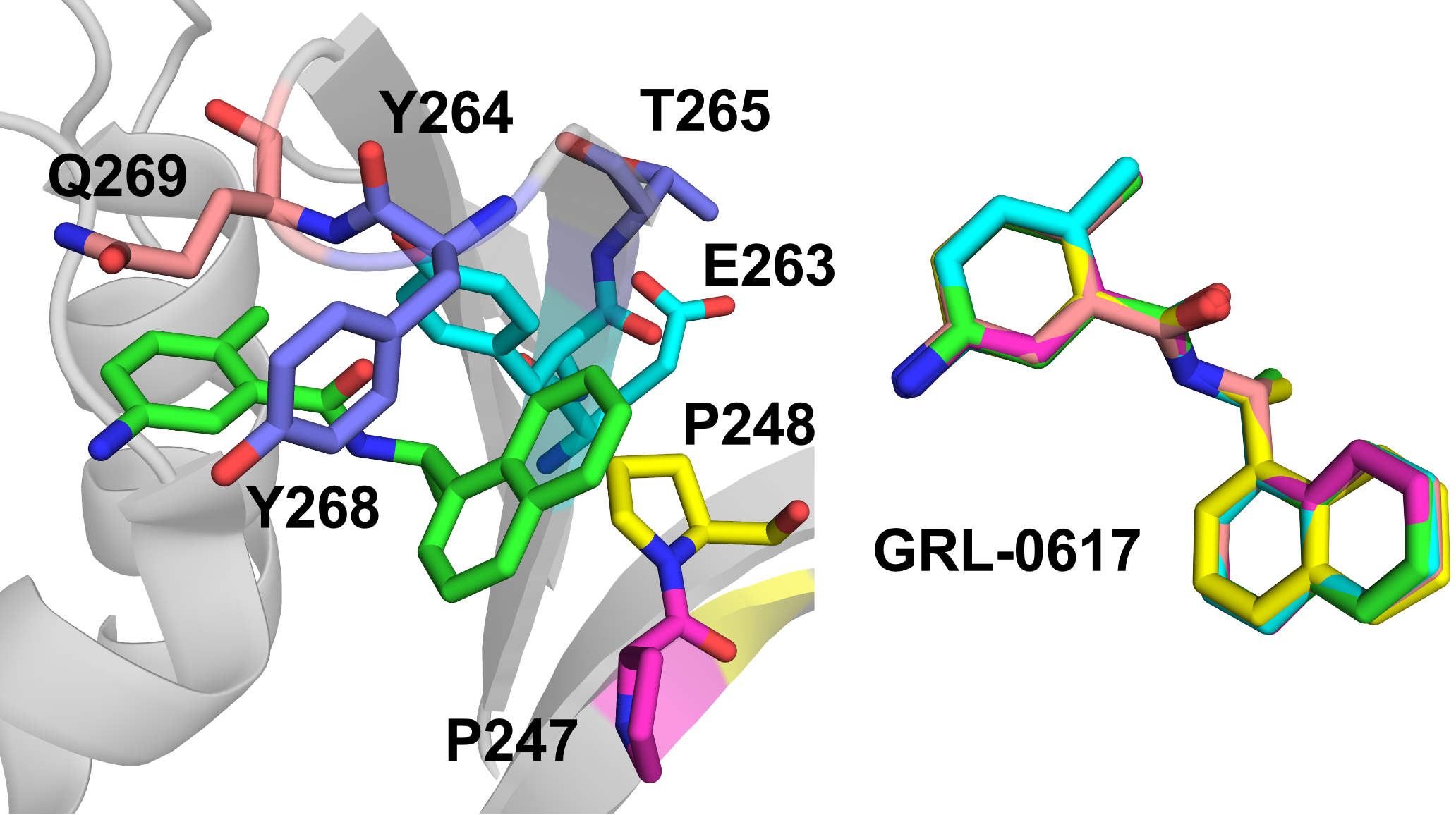

Supplement: S6 Fig — Left: Location of amino acids subject to mutation relative to the ligand (GRL-0617) binding site (based on PDB ID: 7jrn). Right: Representative ligand conformations bound to PLpro. Based on clustering of ligand conformations from Molecular Dynamics trajectories (sampled every 100 ps). (TIF) [file pcbi.1010667.s006.tif]

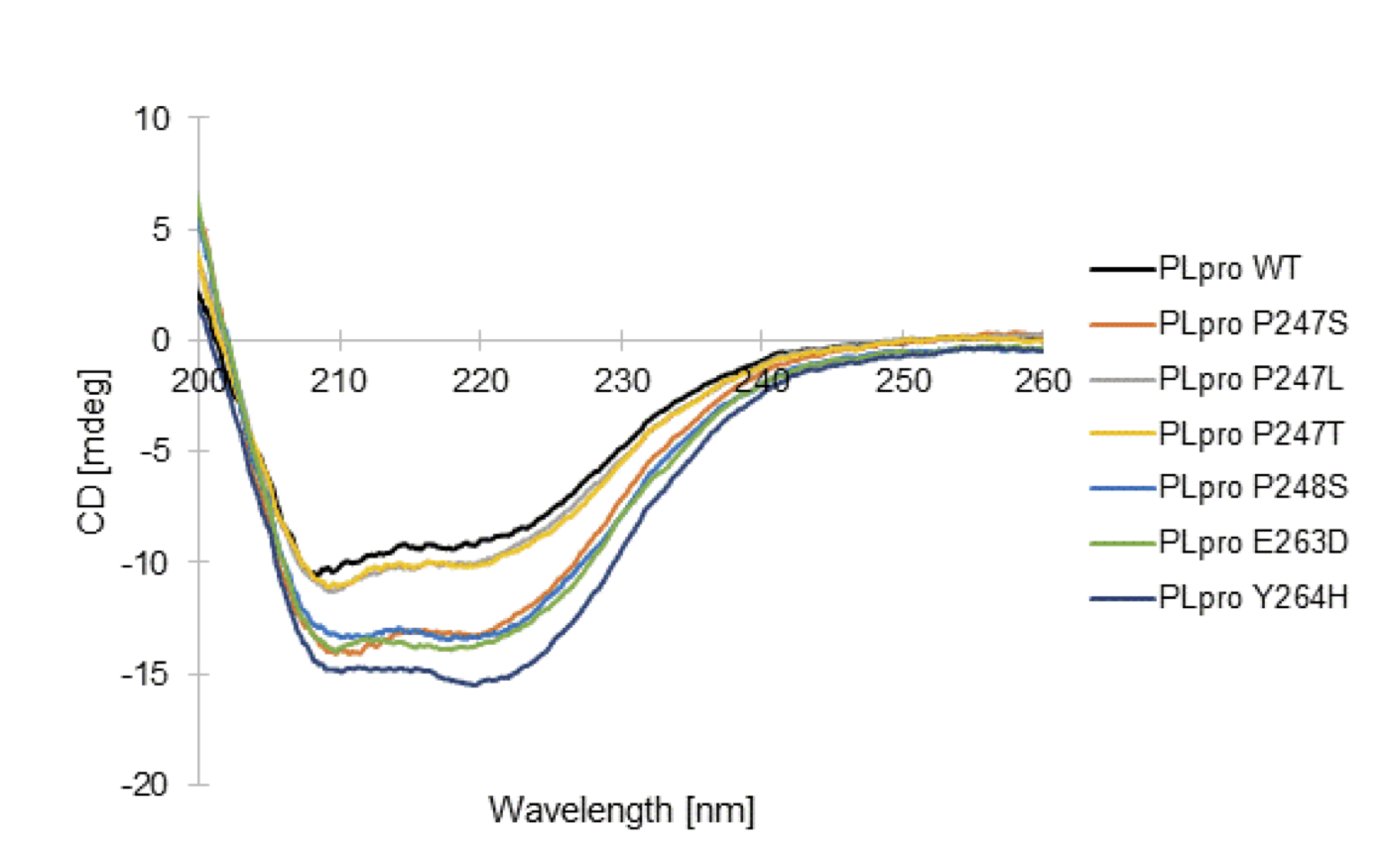

Supplement: S7 Fig — Circular dichroism (CD) measurements for wild type of PLpro protein and its variants displayed a spectrum which shows negative ellipticity between 205 and 240 nm. It suggests that all of them may be in native state and have similar to wild type scaffold. (TIF) [file pcbi.1010667.s007.tif]
