# Supplementary material for: Amino acid variants of SARS-CoV-2 papain-like protease have impact on drug binding
Source: PLoS Comput Biol. 2022 Nov 21;18(11):e1010667. doi: 10.1371/journal.pcbi.1010667 (PMC9721480; doi:10.1371/journal.pcbi.1010667)
Supplement: S1 Table — The bolded entries with asterisk indicate the trajectories in which the ligand dissociated from the binding site. For analysis, only the frames with ligand present in the binding site were used. (PDF) [file pcbi.1010667.s008.pdf]

S1 Table: Length of the PLpro-inhibitor trajectories (in ns) analyzed in this study. The bolded entries with asterisk indicate the trajectories in which the ligand dissociated from the binding site. For analysis, only the frames with ligand present in the binding site were used.

**Variant (trajectory)**	**GRL-0617**	**S43**
WT:1	500	500
WT:2	500	500
WT:3	-	500
WT:4	-	500
G163S:1	500	-
A246V:1	500	-
P247Q:1	500	-
P247Q:2	500	-
P247S:1	500	**5.5***
P247S:2	500	500
P247S:3	-	500
P247S:4	-	**44***
P248S:1	407	500
P248S:2	500	500
P248S:3	500	-
E263D-Y264H:1	**194***	500
E263D-Y264H:2	**370***	**415***
Y264H:1*	**330***	-
Y264H:2	500	-
N267D:1	500	-
T265A-Y268C:1	**201***	500
T265A-Y268C:2	500	500
T265A-Y268C:3	500	**426***
Q269R:1	500	500
Q269R:2	500	500
