# Supplementary material for: Amino acid variants of SARS-CoV-2 papain-like protease have impact on drug binding
Source: PLoS Comput Biol. 2022 Nov 21;18(11):e1010667. doi: 10.1371/journal.pcbi.1010667 (PMC9721480; doi:10.1371/journal.pcbi.1010667)
Supplement: S2 Table — Crystal—data for PDB ID: 7d7t with information on distance between rings. For trajectories—percentage of trajectory in which interaction occurs (i.e. D < 5Å; based on frames every 100ps). Trajectories in which the ligand dissociated from the protein are indicated with *. A1, A2—naphthyl group; B—piperidine; C—benzene. (PDF) [file pcbi.1010667.s009.pdf]

S2 Table: Hydrophobic interactions between S43 ligand rings and protein amino acids. Crystal – data for PDB ID: 7d7t with information on distance between rings. For trajectories – percentage of trajectory in which interaction occurs (i.e. D < 5Å; based on frames every 100ps). Trajectories in which the ligand dissociated from the protein are indicated with *. A1, A2 – naphthyl group; B – piperidine; C – benzene.

	A1- -P247	A1- -P248	A1- -Y264	A1- -Y268	A2- -P247	A2- -P248	A2- -Y264	A2- -Y268	B- -Y264	B- -Y268	C- -K157	C- -Y268	C- -Q269
Crystal	-	4.8Å	-	4.8Å	4.7A	4.8Å	-	-	-	4.5Å	-	-	-
WT:1	0.2	68.7	0	24.2	17.3	81.3	0	13.8	69.6	40.5	0.6	1.4	-
WT:2	0.1	74	0	24.9	24.7	82.3	0	18	59.3	53.7	1.5	1.5	-
WT:3	0.2	75.2	0	26.9	22.6	82	0	18.5	60.2	52.5	0.6	1.8	-
WT:4	0.3	77.4	0	22.8	30	73.2	0	18.1	56.9	48.5	0.8	2	-
E263D-Y264H:1	0.4	52.8	4.3	21.5	7.3	84.7	0	19.2	10.4	34.6	19.6	1.4	-
E263D-Y264H:2*	0.2	60.7	5.5	22.1	9.9	85.3	0	19.2	12.4	29.5	38.2	0.6	-
T265A-Y268C:1	0.4	49.5	0.2	37.6	4.4	34.8	0	18	36.9	7.1	1.4	0.9	-
T265A-Y268C:2	2.5	63	0	42.9	42	73.3	0	11.4	32.3	14.7	48.3	0.6	-
T265A-Y268C:3*	0.2	63.8	0.4	48.4	6.8	58.3	0	17	48.8	12	2.2	2	-
P247S:1*	-	85.7	0	7.1	-	67.9	0	28.6	60.7	19.6	0	8.9	-
P247S:2	-	66.9	0	24	-	70	0	12.6	53	38.2	5.8	1.4	-
P247S:3	-	75.4	0	25.1	-	78.7	0	12.6	48.1	41.9	11.3	1.6	-
P247S:4*	-	64.4	0	4.5	-	68.9	0	3.6	46.2	7.5	4.3	5.7	-
P248S:1	0.9	-	0.2	17.3	14.2	-	0	22.7	63.4	36.5	2.7	1.4	-
P248S:2	0.4	-	2.5	12.9	2.6	-	0.3	16.2	57	27.1	6.6	1.5	-
Q269R:1	0	64	0	21.2	12.9	74.6	0	10.5	66.4	33	1.3	0.6	42.4
Q269R:2	0.1	68.7	0	25.9	25	81.9	0	13.4	76.8	41.8	12	0.4	48.3
