# Supplementary material for: Amino acid variants of SARS-CoV-2 papain-like protease have impact on drug binding
Source: PLoS Comput Biol. 2022 Nov 21;18(11):e1010667. doi: 10.1371/journal.pcbi.1010667 (PMC9721480; doi:10.1371/journal.pcbi.1010667)
Supplement: S3 Table — Crystal—data for PDB ID: 7jrn with information on distance between rings. For trajectories—percentage of trajectory in which interaction occurs (i.e. D < 5Å; based on frames every 100ps). Trajectories in which the ligand dissociated from the protein are indicated with *. A1, A2—naphthyl group; B—4-methylbenzenamine moiety. (PDF) [file pcbi.1010667.s010.pdf]

S3 Table: Hydrophobic interactions between GRL-0617 ligand rings and protein amino acids. Crystal – data for PDB ID: 7jrn with information on distance between rings. For trajectories – percentage of trajectory in which interaction occurs (i.e. D < 5Å; based on frames every 100ps). Trajectories in which the ligand dissociated from the protein are indicated with *. A1, A2 – naphthyl group; B – 4-methylbenzenamine moiety.

	A1- -P247	A1- -P248	A1- -Y264	A1- -Y268	A2- -P247	A2- -P248	A2- -Y264	A2- -Y268	B- -Y264	B- -Y268	B- -Q269
Crystal	-	4.5Å	-	-	-	5.0Å	-	5.0Å	-	-	-
WT:1	0.1	82.2	0.2	8	4.4	61	0	22.6	0	5.5	-
WT:2	0.2	81.4	0.3	8.4	4.2	63	0	24.6	0	6.7	-
G163S:1	0.1	81.1	0.1	7.7	2	62.3	0	21.2	0	7.6	-
A246V:1	0	86.5	0.1	5.4	2.5	54.7	0	31.6	0	6.6	-
P247S:1	-	78.9	0.3	7.6	-	62.7	0	22.6	0	6.5	-
P247S:2	-	80.8	0.1	7.5	-	63.1	0	20.7	0	6.5	-
P247Q:1	-	77.1	0.2	7.6	-	56.5	0	24	0	6.1	-
P247Q:2	-	80.3	0.6	7.5	-	65.5	0	29.9	0	6	-
P248S:1	0.4	-	0.2	5.11	0.9	-	0	44.8	0	4.5	-
P248S:2	0.2	-	0.2	2.4	1.7	-	0	40.1	0	3.8	-
P248S:3	0.1	-	0.1	1.9	0.4	-	0	41.2	0	4.3	-
E263D-Y264H:1*	0.1	74.4	1.1	10.9	12.8	66.5	0	43.8	0	9.7	-
E263D-Y264H:2*	0.1	74.5	4.1	15.1	7.5	73.1	0	40.7	0	8.4	-
Y264H:1*	0.8	73.2	0.5	13.9	3	41.2	0	53.4	0	4.1	-
Y264H:2	0.1	63.7	2	9	1.5	43	0	46	0	8.5	-
Y264H:3	0.3	81.6	1.2	15.6	11.4	62.3	0	53.6	0	5.6	-
Y264H:4	0.1	73.3	3.5	9.2	3.5	56.8	0	45.1	0	9	-
T265A-Y268C:1*	0.3	64	1.7	70.5	3	53.6	0	47.5	0	35.7	-
T265A-Y268C:2	0.1	70.8	4.2	83.1	3.4	48.4	0	74	0	37.5	-
T265A-Y268C:3	0.4	68.3	1.8	76.6	2.3	55.6	0	58	0	29.8	-
N267D:1	0	80	0	6.7	1.9	62.3	0	20.2	0	6.5	-
Q269R:1	0	79	0.2	7.8	2.5	55	0	24.7	0	5.2	43.6
Q269R:2	0.1	79.4	0.1	6	1.5	53.5	0	24.8	0	5.6	44.9
