# Supplementary material for: Amino acid variants of SARS-CoV-2 papain-like protease have impact on drug binding
Source: PLoS Comput Biol. 2022 Nov 21;18(11):e1010667. doi: 10.1371/journal.pcbi.1010667 (PMC9721480; doi:10.1371/journal.pcbi.1010667)
Supplement: Attachment — Submitted filename: letter.pdf [file pcbi.1010667.s013.pdf]

Dear Editor,

We would like to thank you for your letter and the comments from reviewers about our manuscript. We have carefully checked our manuscript according to the comments. The point-by-point response to the comments follows. (Our answers are shown in red).

Reviewer #1: The manuscript 'Amino acid variants of SARS-CoV-2 papain-like protease have impact on drug binding' by Perlinska et al. presents a study on the effect of mutations on the binding mode of potential inhibitors to the Papain-like Protease (PLP) of SARS-CoV2. The study combines Molecular Dynamics (MD) simulations with molecular docking, sequence analysis, MMPBSA calculations and experimental enzyme activity assays to characterize the impact of five mutations: P247S, E263D-Y264H and T265A-Y268C on the affinity of potential non-covalent inhibitors of PLP.

The manuscript is well-structured and all methods that have been used in this study are described in detail. The authors' conclusions are supported by the results, although this referee has some remarks on the methodology used in their study (see below). This referee recommends this manuscript to be published after a minor revision.

In the following, I will point out my remarks that appeared to me during the study of this manuscript:
1. Sequence redundancy in the selected dataset:
The selected data of PLP-sequences seems to be highly redundant, which means that the sequence-similarity among a large fraction of the selected CoV-2 datasets seems to be redundant as well. To remove artefacts that arise from sequence-redundancies, only sequences with a similarity that lies below a certain threshold should be considered < 80 %. (see the mutation rates in Table 1).
The mutation-rates can be reflected using a position-dependent heat-mapped color-coding on Figure 1.
We agree that the dataset is highly redundant - in fact we found that a lot of the sequences are identical with the reference. However, since we seek single point mutations (variants) we needed to ensure that we do not filter out the sequences that differ even on one position. In fact, to acknowledge all the new information that appeared after our first analysis we downloaded current set of ORF1a sequences (2,7 mln) and redone the analysis. Now we see that 21% of them contain variants (either single or multiple).

2. Median of MMPBSA-interaction energies :
The median values and the variances of the measured energies (see Figure 3 D) indicate that there is no evident difference in the energies between the mutational variants and the wild-type, because the error-bars are crossing each of the individual median values.
A running averaging that also considers the statistical error might be more indicative for the energy differences : 100 ps, -> av_2 1 ns -> av_3 10 ns -> av_4 100 ns -> av_5 500 ns (final result) + the statistical error.
As MMPBSA is a quite inaccurate method for the calculation of interaction energies, the study could be improved using thermodynamic integration (TI) or Free energy perturbation (FEP) calculations.

Thank you for the suggestion. We recalculated the averages and errors using block averaging that resulted in more distinctive differences between the mutants and wild type energies. We added this paragraph to the Methods:
"The averages and standard errors shown on Figure 3D are calculated using block averages. For each trajectory we obtained the standard error of the average based on trajectory segments of length *n* (blocks). For each *n* we calculated first the standard deviation among the block averages (σ_n) and then the standard error: $\sigma_n/\sqrt{M}$, where M is the number of the blocks of length *n*. Then, for each trajectory we chose *n* based on the plateau of the standard error."

3. The RMSDs of the residues in the drug-binding pocket over time can be added for each mutational variant, as this metric might be an indicator for the stability of the drug-binding site compared to the wild-type. Further, it might explain the differences in the enzymatic activity that has been observed experimentally.
We performed RMSD calculation of the residues interacting with the ligands and on average the difference between WT and mutants is not significant. Overall, the whole structures of the mutants are behaving in a similar fashion to WT. We added a comment about this to the main text.
Reviewer #2: In this manuscript, the authors tried to clarify different sequences of PLpro and then studied the influence of these mutations on the binding process of ligands to PLpro via docking and MD simulations. In vitro works were then performed to validate the observation. It is of great interest to read the manuscript. A large work was completed, however, there are some comments to improve the manuscripts.
1. The structural change of the PLpro under the effect of mutation would significantly impact the binding free energy and binding pose of ligands to PLpro. So, the MM/GBSA calculation should be carried out over the equilibrium snapshots of the complex, which were obtained from MD simulations instead of molecular docking only.
We agree that the mutation of specific PLpro residues could lead to structural changes with diverse levels of significance, depending on the residue. Naturally, the utilization of multiple PLpro conformations based on MD snapshots would increase the accuracy of the predictions. However, such an approach would also significantly increase the required computational time. Overall, the selection of methods is a matter of strategy and should serve the achievement of goals set for each part of the study.
Herein, we decided to start with fast methods with limited accuracy – docking and MM-GBSA binding energy calculations for single protein-ligand complexes obtained from docking on residue mutations. Then, we moved to more accurate methods – MD with subsequent MM-GBSA and MM-PBSA. Utilization of MD snapshots from the beginning would increase accuracy but would be not effective in terms of accuracy to time ratio. The whole point of the initial MM-GBSA for single PLpro conformations is to avoid conducting an excessive number of MD simulations. The multi-step approach proposed by us provides the best of both worlds – fast, effective filtration at the early stages and an accurate prediction at the latter phases.
That said, we are aware that utilization of methods with such limited accuracy at the beginning of the study could be risky in terms of obtaining both false positive and false negative results. In the case of encountering false-positive results, the latter MD verification is an obvious way to filter out such non-important mutations. We were more concerned about potentially losing some considerable substitutions because of labelling them as false negatives by the methods used at the beginning if the study. For this reason, we

implemented mild cutoffs for scoring function values and estimated binding energies and selected also moderately promising mutations for MD verification.

2. According to the previous comment, the MM/GBSA calculation over docking simulation probably does not make sense since the obtained results are not significantly different eg. the binding energy range from -27.4 +/- 6.1 to -32.28 +/- 5.7 (line 286), or -28.1 +/- 3.6 to -29.0 +/- 3.5 kcal/mol (line 314 - 315), etc. The obtained results are not different within the error bar, authors may wish to discuss about this.
We recalculated the averages and errors using block averaging that resulted in more accurate differences between the mutants and wild type energies. We added this paragraph to the Methods:
"The averages and standard errors shown on Figure 3D are calculated using block averages. For each trajectory we obtained the standard error of the average based on trajectory segments of length *n* (blocks). For each *n* we calculated first the standard deviation among the block averages (σ_n) and then the standard error: $\sigma_n/\sqrt{M}$, where M is number of blocks of length *n*. Then, for each trajectory we chose *n* based on the plateau of the standard error."

3. The MM/GB(PB)SA calculation (Per-residue free energy decomposition) should be carried out since the obtained results will clarify the contribution of each residue of PLpro. The interaction picture would thus be a clarifier.

We performed per-residue decomposition of MMGBSA calculated energy and included it in the supplement as Figure 5. On the figure we show the contribution of selected amino acids from the binding site, those that have the biggest influence on the ligands.

4. The MMGBSA terms should be reported in the manuscript.

The MM-GBSA binding energies calculated from the MD trajectories included terms dependent on the AMBER algorithms. We used salt concentration of 0.1M and igb=5. Overall, the MM-GBSA terms regarding the MD part of the study might be described using the following simplified formulas:

$$\Delta G_{binding} = G_{protein\text{-}ligand} - G_{protein} - G_{ligand}$$
$$G = \Delta G_{el} + \Delta G_{nonel} + \Delta E_{MM}$$

where ΔE_{MM} is the interaction energy in gas phase, and ΔG_{el} and ΔG_{nonel} correspond to polar and nonpolar components. The detailed formulas for the specific terms are available in the freely accessible Amber 2018 Reference Manual (https://ambermd.org/doc12/Amber18.pdf). Note, that the entropic contribution was not calculated because of high computational costs involved with the number of frames we performed the calculation for.

The MM-GBSA binding energy for docking complexes, performed in BIOVIA Discovery Studio, includes the following terms:

$$G = E_{bond} + E_{el} + E_{vdW} + G_{pol} + G_{np}$$

$E_{bond}+E_{el}+E_{vdW}$ are based on CHARMM force field, whereas $G_{pol}+G_{np}$ correspond to GBSA solvation model. The entropic term was not selected in the Calculate binding energies

protocol as it was computationally too costly for this stage of the study. Detailed information regarding the energy terms is not provided by BIOVIA.

Once again, we would like to thank you all for the suggestions and e orts on our manuscript. The culmination of your help made it a much stronger manuscript.

Yours sincerely,
Joanna Sulkowska
